# Can pin-site inflammation be detected with thermographic imaging? A cross-sectional study from the USA and Denmark of patients treated with external fixators

**DOI:** 10.2340/17453674.2024.41901

**Published:** 2024-09-23

**Authors:** Marie FRIDBERG, Ole RAHBEK, Hans-Christen HUSUM, Bafor ANIREJUORITSE, Kirsten DUCH, Christopher IOBST, Søren KOLD

**Affiliations:** 1 Interdisciplinary Orthopedics, Aalborg University Hospital, Denmark; 2 Nationwide Children’s Hospital, Columbus, OH, USA; 3 Research Data and Biostatistics, Aalborg University Hospital, Aalborg, Denmark; 4 Center of Rheumatic Research (CERRA), Aalborg University Hospital, Aalborg, Denmark

## Abstract

**Background and purpose:**

Patients with external fixators are at risk of pin-site infection. A tool for objective monitoring of pin sites for evolving signs of infection is warranted. We aimed to investigate the temperature (MaxTp) difference between clean and visually inflamed pin sites using thermography and to establish the optimal cut-off value of MaxTp using thermography as a screening tool for inflammation detection.

**Methods:**

This was a cross-sectional study performed in the USA and Denmark of patients with circular external fixators. Pin sites were visually judged by a surgeon or a nurse as clean or as showing signs of inflammation. The MaxTp was obtained at the pin site by thermographic imaging using an infrared camera (FLIR T540).

**Results:**

We included 1,970 pin sites from 83 patients. The mean MaxTp for clean pin sites (n = 1,739) was 33.1°C (95% confidence interval [CI] 32.8–33.4) and the mean MaxTp for visual inflamed pin sites (n = 231) was 34.0°C (CI 33.6–34.3). The mean difference, when adjusted for repeated observations of patients and pin sites, was statistically significant with a difference of 0.9°C (CI 0.7–1.1) (P < 0.001). The area under the receiver operating characteristic curve for MaxTp as a screening tool to detect visual signs of inflammation was 0.71 (CI 0.65–0.76). The empirically optimal cut-off value was 34.1°C with a sensitivity of 65%, a specificity of 72%, a positive predictive value of 23%, and a negative predictive value of 94%.

**Conclusion:**

We found a statistically significant difference in mean temperature between pin sites with and without visual signs of inflammation. Thermography could be a promising tool for future point of care technology for monitoring inflammation around pin sites.

Patients undergoing treatment with external fixators are at risk of developing a pin-site infection [1,2]. Most pin-site infections are superficial and can be treated with increased pin-site cleaning or oral antibiotics [3]. However, if not recognized early, deep soft-tissue infection or osteomyelitis may occur, which can compromise the outcome due to pin loosening, frame instability, or the need for surgical removal of hardware. The incidence of pin-site infection has been reported to be 1–100% [1-5], but the true incidence of pin-site infection is not known, as no universal definition of a pin-site infection exists [6]. In 2012 a Cochrane review by Lethaby et al. [7] concluded that no strategy for the best management of pin sites could be recommended due to insufficient evidence of adequate quality. This non-consensus on pin care management was confirmed in 2022 by 2 systematic reviews [8,9] initiated by the International Pin Site Consensus Group [10].

Currently, the clinical decision-making process of possible pin-site infection is subjective and consists of visible signs of inflammation combined with the patient’s subjective sensation of pain and irritation [11]. Nonspecific, time-consuming laboratory tests such as blood counts and microbiology might be used for decision support and monitoring as well. We believe, as do others [3], that the development of pin-site infection is a continuum proceeding from a non-inflamed pin site to inflammation and eventually infection. However, a more objective assessment of possible pin-site infection is warranted, particularly for future home-based monitoring of pin sites. A proof-of-concept study [12] on a small number of pin sites showed that thermography might have the potential to detect inflammation at the pin site. As smartphones with integrated thermal cameras are now available, thermography might be used as a home-based surveillance tool [13].

The aim of our study was to investigate the temperature (MaxTp) difference, as measured by thermography, between clean and visually inflamed pin sites. Second, we established the optimal cut-off value of MaxTp using thermography as a screening tool for inflammation detection.

## Methods

### Study design

This was a cross-sectional study. Reporting follows the STROBE guidelines for cross-sectional studies.

### Setting

The study was conducted at Aalborg University Hospital, Denmark (Aalborg UH) from April 21, 2021, to April 13, 2023 and at Nationwide Children’s Hospital, Columbus, Ohio, USA (NCH) from February 1, 2022 to November 14, 2022.

### Participants

Eligibility criteria were all patients surgically treated with a circular external fixator who had undergone surgery more than 14 days prior to a scheduled visit to the orthopedic outpatient clinic at Aalborg UH or NCH. Exclusion criteria were patients not wanting to participate and pin-site examinations where the temperature measurements were missing or unobtainable due to incorrect camera settings or focus issues, or if the ambient room temperature or the image was missing. Recruitment of patients was done on weekdays when thermographic images could be obtained by a research staff member. Informed consent was obtained from the patient or, for minors, from the parent prior to inclusion. Patient-reported demographic data and etiology for treatment were collected at inclusion. The Fitzpatrick Skin Phototype Classification 0–6 scale (FSPC) was used to evaluate the skin phototype (Type 1 is light, pale white Type 6 is very dark, brown to black) [14]. All pin sites were labelled with a unique pin number. For each pin site it was noted whether the pin site was a transfixion wire or a half pin, and its anatomical location (foot, lower leg, or thigh) was registered.

### Data sources and variables

***Visual signs of inflammation.*** The clinical visual grading system the Modified Gordon Score (MGS) classification system was used to assess all pin sites for signs of inflammation and infection on a 0 to 4 scale (Table 1) [8,10]. Because the aim was to differentiate clean and inflamed pin sites, the dataset was based on the MGS score secondarily divided into 2 groups of clean (MGS = 0) and inflamed (MGS > 0). MGS 0 describes a clean pin site with no erythema and no discharge. MGS > 0 describes pin sites with visual signs of inflammation and infection (Table 1).

**Table 1:** The Modified Gordon Score (MGS) pin site infection classification system

MGS grade	Visual description
0	Clean
1	Serous drainage, no erythema
2	Erythema, no drainage
3	Erythema and serous drainage
4	Erythema and purulent drainage (pus)

Grade 2–4 erythema is judged as a clinical redness suspicious of inflammation (a continuum with signs of infection) in contrast to redness from scarring around the pin site, which is not graded as erythema. The nurse/surgeon need to ask/look for pins with drainage today to do the MGS score. If the pin is dry today the score is either 0 or 2

The reason for using the MGS classification [15] was based on the idea of using a grading scale that covers only visible signs of inflammation and infection. There is no universally accepted definition of a pin-site infection and previously used clinical classification systems all lack reliability [6,11,16]. We have addressed this issue by incorporating an investigation of the reliability of the MGS into this current study.

***Thermographic examination.*** For thermographic imaging, an infra-red (IR) thermal camera was used (FLIR T540, FLIR Systems AB, Täby, Sweden) with an integrated digital camera. Auto-focus was obtained by pointing the center of the IR detector on the pin, which is a perfect contrast spot having both the cold pin and the warm skin in the field of view. Prior to thermographic examination, the patient’s legs and feet were exposed and the patient was positioned on an examination table. The patient remained in this position for 10 minutes to allow the skin to acclimatize to the ambient room temperature. Ambient room temperature was recorded as the photo session started. Dressings were removed before the 10 minutes of acclimatization. No pin care was done until the photo session was completed.

**Figure 1:**
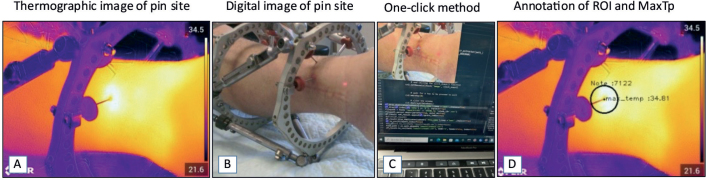
(A) Thermogram focused on a pin site with MGS grade 3. (B) Digital image of the same pin site (MGS grade 3). (C) The one-click method with only the corresponding digital image without a heatmap visible for the examiner, to avoid examiner bias during the process of extracting the pin site temperature from the thermograms. (D) ROI outlined at the pin site, annotated using the one-click method with a fixed ROI size. ROI = region of interest, MGS = Modified Gordon Score pin site infection classification system, MaxTp = maximum temperature within the region of interest.

Each pin site was then examined with the thermographic camera, and an IR image and digital image were captured simultaneously (Figure 1). A digital imprint of the pin number and study ID was recorded. The photographer was instructed to have 40–60 cm distance from the skin surface to the camera lens and to obtain the image with the most possible visible skin around the pin, with the pin site centered and an angle of approximately 45–90° to the skin surface. It was ensured that the frame construct did not obstruct the view of the pin site area. Consistency of the distance between the camera lens and the pin site was ensured by the camera’s continuous laser distance measurement function. The emissivity setting was always at 0.98 as recommended for human skin [17,18]. All camera specifications and settings are outlined in Table 2. The photographer was any research staff member (surgeon, orthopedic nurse, research assistant) who was available on the day and had informally been trained by and received instructions from the first author.

**Table 2:** Settings, accuracy test, and specifications of infrared cameras used

Infrared camera	Manufacturer: FLIR Model: T540
Last equilibration	Alborg UH December 8, 2020.
by manufacturer	NCH July 18, 2019
Black box test of precision	Aalborg UH 0.2°C.
	NCH 0.6°C (above absolute)
Resolution	464 x 348 pixel (total 161,472)
Focus	Continuous auto/manual contrast
Spectral range for detector	Uncooled microbolometer/7.5–14 *µ*m
Lens	17 mm/24°
Field of view	24° × 18°
Sensitivity (thermal)	< 0.03°C (@ 30°C)
Emissivity ^a^ setting	0.98
Ambient temperature	23°C
Distance	Continuous laser assisted (40–60 cm)
Target angle	90–45°
Temperature range	–20 to +120°C
Accuracy (manufacturer)	±2°C (for ambient temperature 15–35°C and object temperature above 0°C )
Reflected temperature	30°C
Humidity	50%
Spatial resolution (IFOV)	0.90 mrad/pixel
NETD	< 40 mK @+30°C (+86°F)

a Emissivity is a physical constant (E) defined as the ratio of the energy radiated from a material’s surface to that radiated from a perfect emitter (a black body) at the same temperature, wavelength, and under the same conditions. The emissivity of skin is 0.97–0.99 [35].

NETD = Noise equivalent temperature difference or thermal sensitivity is a measure for the thermal sensor’s ability to distinguish small differences in infrared radiation in the image.

IFOV = Instantaneous field of view or spatial resolution is a measure that characterizes the infrared sensor and refers to the distance represented by a pixel in an image.

### Analyzing the thermal images

The thermograms were transferred to a computer and the temperature measurements at each pin site were extracted using a one-click method based on a custom-built Python tool [19]. A fixed-size circular region of interest (ROI) was placed around the pin, with the center positioned manually exactly where the pin passes through the skin (ROI: 30 pixel radius, area of 2,809 pixels). The maximum temperature measured within the ROI (MaxTp) was used as the primary outcome measure (Figure 1). All images were analyzed by 1 of 2 different examiners, (a) a medical student and (b) the senior first author.

## Bias

The center of ROI was manually placed by 1 click on the digital image without a heatmap, which blinded the examiner to the thermogram colors revealing the temperature pattern.

The accuracy of both cameras used in this study were tested against an ISO-certified equilibrated black box to avoid systematic bias from the precision of the infrared detectors. The factory standard for the cameras was an absolute accuracy of ±2 °C, but they proved more precise with a consistent variation of the accuracy of 0.1 °C between the 2 cameras.

To examine for any detection bias in the MGS classification of pin sites, stemming from some pin sites being graded by an orthopedic nurse, a sample of 1,127 pin sites at Aalborg UH received double grading by both the orthopedic surgeon and the orthopedic nurse. There was excellent agreement in classifying the pin sites as clean and showing signs of visual inflammation (Kappa = 0.85, SE 0.3).

To assess the inter-observer reliability of the 1-click method for temperature measurement, MaxTp was extracted from a random sample of 80 thermograms by the 2 examiners. There was near-perfect agreement in the extracted temperature measurements (ICC = 0.99, CI 0.99–0.99).

### Study size

Sample size estimation was based on obtaining reasonable precision when estimating the area under the receiver operating characteristic (ROC) curve based on the formula from Cortes and Mohri [20]. The estimated standard error of the area under the curve (AUC) value was set to 0.05 (corresponding to a deviation of the AUC value by 2 decimal points), the positive rate to 0.1, and the error rate to 0.1. Assuming the pin-site observations were independent, the sample size needed was 380. Due to the cross-sectional study design, we expected that patients would be included more than once on different days with repeated non-independent observations, and we decided to include 2,000 observations to ensure sufficient observations.

### Statistics

Discrete variables are summarized as counts and percentage, while continuous variables are presented as mean and standard deviation (SD), or median and interquartile range (IQR), or range as (min, max) as appropriate.

The least-square means of MaxTp of pin sites categorized as clean and those displaying visual signs of inflammation are presented for each group. The mean MaxTp difference between the 2 groups with accompanying 95% confidence interval (CI) is presented. Significance testing was performed using an unpaired 2-sample Student’s t-test.

To evaluate the performance of thermography as a screening tool for inflammation detection, with inflammation defined as an MGS score > 0, an ROC curve based on probabilities from logistic regression was plotted, and the area under the ROC curve (AUC) is presented. Youden’s empirically optimal cut-off value of MaxTp, and associated sensitivity and specificity, is presented [21].

In a violin plot the mean MaxTp distribution of clean and inflamed pin sites is presented. Median, IQR, and outliers were calculated for each plotted group.

***Sensitivity analysis.*** The study design allowed for multiple examinations per patient on different dates (dependent observations). A mixed model was used to analyze the mean temperature difference between clean and inflamed pin sites while adjusting for bias introduced by repeat observations of patients and individual pin sites as random effects. To account for any effect of dependency between observations, ROC analysis with 1,000 bootstrap repetitions clustered by patient ID was performed. No difference in standard error or CI was detected.

***Missing data.*** Missing data was identified during the process of analyzing the thermograms due to incorrect camera setting or blurred focus (Figure 1). If a pin site was missing any data, that specific pin site was excluded from statistical analysis.

Statistical analyses was performed using Stata version 18.0 (StataCorp, College Station, TX, USA).

### Ethics, data sharing, funding, use of AI, and disclosures

The protocol was registered at Region Nord Jylland, Danish Data Protection Agency (File: 2021-05), and approved by the Danish Local ethical committee (File:77289) and NCH IRB approval (STUDY00002178). Data Use Agreement For the Transfer of Data was fulfilled (ID CBJCHBCAABAAUxriGOMDs8eC5WZ0o0MxamSlAZ0fHWT2). Funding was received from the Novo Nordisk Foundation (File: NF 20OC0065770), Innovation Foundation of Region of Northern Denmark (File: 2020-006248), Kopps Foundation, and Toyota Foundation. None of the funding has influence on the reporting and data collection. Artificial intelligence was not used in any steps of analyzing data or writing the manuscript. None of the authors have any conflicts of interest. Complete disclosure of interest forms according to ICMJE are available on the article page, doi: 10.2340/17453674.2024.41901

## Results

### Patients and recruitment

**Figure 2:**
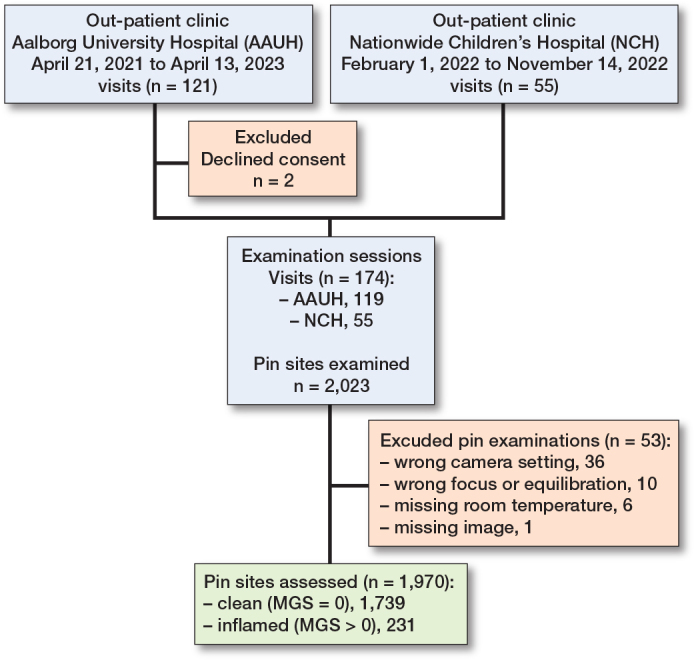
Consort diagram: study flow of participants located at Aalborg UH and NCH. Number of visits, patients, examinations, and pin sites at each step. MGS = Modified Gordon Score pin site infection classification system.

We screened 85 patients for eligibility of whom 83 consented. In total 174 examination sessions were conducted with demographic data collection and clinical assessment of 2,023 pin sites (Figure 2). At Aalborg UH the pin sites were assessed by an orthopedic surgeon and an experienced orthopedic nurse using the MGS (n = 1,127). If the surgeon was unavailable only the nurse assessed the pin sites (n = 341). At NCH-US, an orthopedic surgeon scored all pin sites using the MGS (n = 555). Missing data were identified from 53 pin sites. The reasons for exclusion were incorrect camera setting (36 pin sites), focus and equilibration issues (10 pin sites), missing ambient room temperature measurement (6 pin sites), and missing thermographic/digital image (1 pin site). After exclusion, data from 1,970 pin sites, of which 559 were non-repeated observations, collected from 83 patients at 168 examination sessions, were included for analysis. From the visual grading we identified 1,739 (88%) categorized as clean pin sites and 231 (12%) pin sites displaying signs of inflammation. This division was done from MGS classifications available for 1,629 pin sites judged by a surgeon and for 341 pin sites judged by the nurse. The median number of examinations per patient was 1.0 (range 1–18). The median interval between surgery and examination was 69 (range 14–408) days. The mean ambient room temperature was 23.5°C (SD 1.5). Demographics, population characteristics, and clinical data are presented in Table 3. Anatomical locations of the pins were for 22 (1%) on the thigh, for 1,674 (85%) on the lower leg, and for 274 (14%) on the foot (Table 3). The total number of pediatric patients included was 34 (defined as age below 18 years) corresponding to 287 wires and 277 pins in the study population.

**Table 3:** Demographics, characteristics, and clinical data

Total number of patients	83
Examinations per patient, median (range)	1.0 (1.0–18.0)
Female/male sex, n	37/46
Age, median (range)	24 (6–88)
BMI, mean (SD)	25.9 (8.4)
Smokers, n	8
Comorbidity, n	
Hypertension	9
Diabetes mellitus type I	3
Diabetes mellitus type II	3
Cardiopulmonary disease	2
Cancer	3
Other ^a^	9
Fitzpatrick skin tone type, n	
1 (light)	26
2	44
3	5
4	3
5	2
6 (dark)	2
Indication for frame treatment, n	
Fracture	40
Correction or lengthening (no prior infection)	33
Malunion/non-union/delayed healing	9
Not specified etiology	1
Frame construct and design, median (range)	
Total number of half-pins/wires per patient	11 (6–25)
Number of half-pins per patient	6 (0–20)
Number of wires per patient	5 (0–8)
Frame design, location of pins and wires, n	
Tibia only	62
Femur/tibia	1
Tibia/foot	19
Femur/tibia/foot	1

a Patient-reported comorbidity: Klinefelter, neurofibromatosis, skeletal dysplasia, fibula hemimelia, hypophosphatemia, Rickets, apoplexy, asthma, scleroderma, Charcot–Marie–Tooth, rheumatoid arthritis, osteoporosis, hypercholesterolemia, thrombocytopenia.

### Temperature and visual signs of inflammation

**Figure 3:**
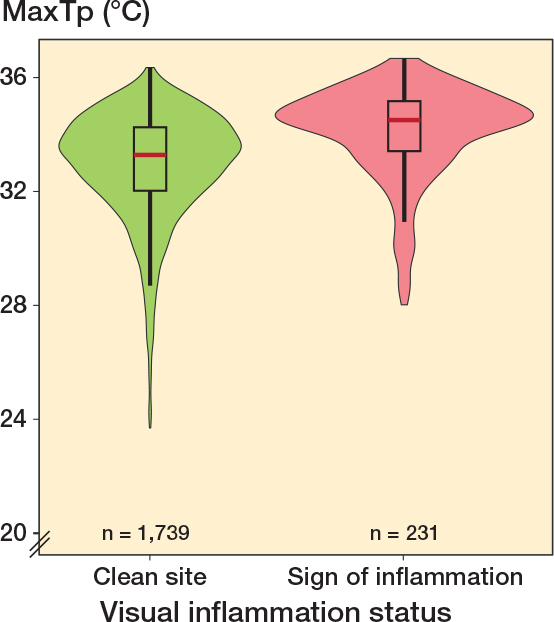
Violin box plot of temperature distribution for clean pin sites (left, green) and inflamed pin sites (right, red). The median, interquartile range (IQR) and outliers are marked for each plotted group. The y axis is MaxTp °C (maximum temperature within the region of interest). Total number of observations = 1,970.

The least square mean MaxTp for clean pin sites (1,739) was 33.1°C (CI 32.8–33.4) and the least-square mean MaxTp for visual inflamed pin sites (231) was 34.0°C (CI 33.6–34.3). The mean difference was 1.2°C (CI 0.9–1.4) (P < 0.001). The mean difference, when adjusted for repeated observations of patients and pin sites, was statistically significant at 0.9°C (CI 0.7–1.1) (P < 0.001). The MaxTp distributions of pin sites judged as clean (MGS = 0) and inflamed (MGS > 0) are visualized in a Violin plot in Figure 3.

### MaxTp cut-off value for detecting visual signs of inflammation

**Figure 4:**
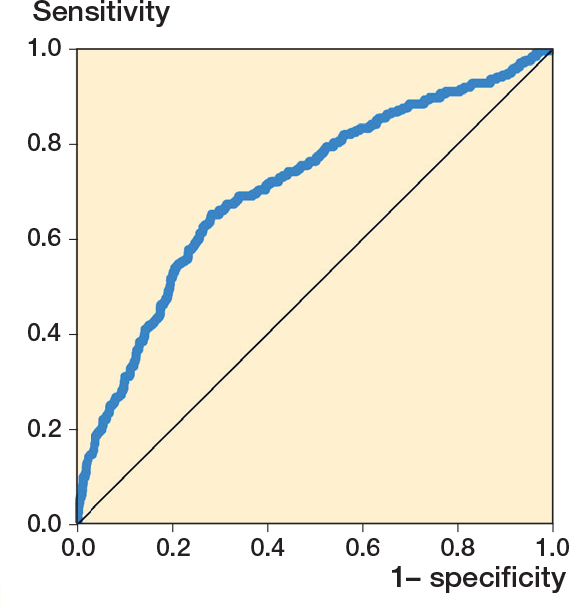
Receiver operator characteristic (ROC) curve for MaxTp (maximum temperature within the region of interest) as a screening tool for inflammation (MGS > 0). Each plotted point represents the true positive rates at the y axis (sensitivity) and the false positive rates at the x axis (1-specificity) at different MaxTp cut-off points. Performance of MaxTp in distinguishing pin sites as being clean or inflamed was the area under the ROC curve (AUC 0.71, CI 0.67–0.75), standard error (0.02). Observations (n = 1,970). MGS = Modified Gordon Score pin site infection classification system.

A ROC curve depicting the performance of MaxTp in distinguishing pin sites as being clean and inflamed (MGS > 0) is plotted in Figure 4. The AUC was 0.71 (CI 0.65–0.76). Adjusting for dependency between the observations by bootstrapping did not significantly affect the associated CI. The empirically optimal temperature cut-off value was calculated to be 34.1°C (sensitivity of 65%, specificity of 72%, positive predictive value of 23% and negative predictive value of 94%) for distinguishing between pins without (MGS = 0) and pins with visual signs of inflammation (MGS > 0).

## Discussion

We aimed to investigate the temperature difference between clean and visually inflamed pin sites using thermography to establish a cut-off value. We found that the sensitivity was 65% and the negative predictive value was 94% of thermography when using 34.1°C as threshold for inflammation. Even though thermography has been suggested as a tool for detecting infection in wounds [22-24], to our knowledge, the only previous study that has examined its use as a potential tool for surveillance of pin-site infection is Rahbek et al. [12]. In this prior pilot study, a cut-off value of 34°C was suggested as the optimum maximum temperature to distinguish between non-infected (MGS = 0) and infected pin sites (MGS > 0) but only 11 pins had MGS > 0 and none had MGS = 4 and therefore the validity of these findings can be questioned. In addition, a low-resolution thermographic camera was used. In the present study we have examined a larger cohort with more pin sites that had visual signs of inflammation and infection. The thermograms were obtained with a higher resolution infra-red camera and we have calculated near-identical temperature cut-off value for distinguishing between pin sites with and without visual signs of inflammation, confirming previous findings.

We found a statistically significant difference of 0.9°C (CI 0.7 to 1.1) in MaxTp between the visually clean and inflamed pin sites (MGS = 0 vs MGS > 0), which is in concordance with other studies exploring the association between inflammation and increased wound temperatures [12,22-27]. A recent scoping review reported that temperature peaks after the initial 2 weeks postoperatively are likely due to infection [28].

The absolute accuracy of the temperature measurements varies with the thermographic camera used. The presented cut-off values should therefore be considered valid only for thermographic cameras with similar accuracy. We would note, however, that this limitation in external validity might not be relevant if pin-site temperatures are followed over time allowing each pin site to be its own control.

The results in our study reflect thermography of pin sites in a majority of patients with pale–light skin tone (85% had FSPC 1–2). Based on previous research, our results might still be externally valid to all skin tones because skin emissivity varies from 0.97–0.99 [17] and skin pigmentation does not affect thermal emissivity [29].

Previous clinical studies using thermography have applied a stringent methodology in a laboratory setup to assure standardized recording conditions [30,31]. This study was deliberately performed in our standard outpatient setting. However, we used a thermographic camera with a very high resolution, and we still report on the methodology regarding equilibration, distance/angulation, ambient room temperature, and anatomical factors in accordance with recommendations from the thermography societies [18,32].

Previous studies using thermography to assess wounds have used either the mean temperature in a selected area of interest or a reference area of the ipsilateral site [22,23,33]. The mean temperature of the ROI was specifically not chosen in this study, because the metal pin at the site is always colder compared with the warmer underlying skin and the metal pin area captured in each image and annotated at the ROI will vary with distance, angulation, and being either a pin or a wire. A reference area was specifically not used in this study as local anatomical variations occur.

### Strengths

In this study we have included patients of all ages, different etiology for treatment, and a variety of comorbidities and skin types. The data in our study originates from almost 2,000 pin sites from 2 different international institutions, which to our knowledge is the largest cohort of pin sites examined with thermography. The study design implies that different raters have performed the assessment of the pin sites using the visual grading system and obtained the thermograms, which adds strength to the generalizability. We believe our study population reflects the general heterogeneous practice in the absence of good evidence for pin-care protocols and regimes.

### Limitations

The cross-sectional design of this study reveals an association between the maximum temperature at the pin site and the visual signs of inflammation and infection, but no causal conclusion can be drawn. The majority of the pins were inserted into the foot and the lower leg in the tibial bone. The results from this study should not be extrapolated to pins or wires inserted in the thigh. Thermophysical factors that influence the infrared radiation are traditionally divided into environmental, individual, and technical [34]. In a clinical setting the inability to control all influencing factors will always remain a limitation as it is impossible to eliminate all potential factors compared with a laboratory setup. However, the differences between non-inflamed and inflamed pin sites have in this study proved to be sufficiently large to overcome the inherent thermophysical limitations.

### Conclusion

We identified a statistically significant difference in temperature measurements between pin sites categorized as clean and those displaying visual signs of inflammation, with a difference of 0.9°C (CI 0.7–1.1). The empirically optimal temperature cut-off value was 34.1°C between clean and inflamed pin sites.

### Perspectives

In perspective, thermography may be a promising tool for a future point-of-care technology for pin-site surveillance. We suggest a next step is to collect longitudinal data from each pin site over time, and to use the mean of previously measured MaxTp as its own baseline and pin-specific control. Furthermore, future studies should examine the feasibility of obtaining thermographic images with sufficient quality of pin sites if pictures are taken by patients, relatives, or home nurses in a home-based surveillance setting. Whether the addition of PROMs (e.g. simple pain score) to home-based surveillance will increase detection rates of inflamed pin sites should also be explored.

## Notes

Conceptualization: MF, OR, SK; methodology: MF, OR, SK; software: MF; validation: MF, OR, SK, HCH; formal analysis: MF, HCH, AB, KD; investigation: MF, AB; data curation: MF, AB, HCH; writing—original draft preparation: MF; writing—review and editing: OR, HCH, AB, KD, CI, SK; visualization: MF, HCH; supervision: OR, CI, SK ; project administration: MF, OR, SK; funding acquisition: MF, OR, SK.

Sowmya Annadatha and Ming Shen are acknowledged for technical support, and Mads Emil Jakobsen, Tina Lyngholm Jensen, and Arash Ghaffari for practical assistance.

Handling co-editors: Bart A Swierstra and Robin Christensen

*Acta* thanks Anna Stefánsdóttir and Ewout S Veltman for help with peer review of this manuscript.
